# β-escin selectively targets the glioblastoma-initiating cell population and reduces cell viability

**DOI:** 10.18632/oncotarget.11784

**Published:** 2016-09-01

**Authors:** Elizabeth Harford-Wright, Nicolas Bidère, Julie Gavard

**Affiliations:** ^1^ CRCINA, INSERM, CNRS, Université d'Angers, Université de Nantes, Nantes, F-44000, France; ^2^ Team SOAP, “Signaling in Oncogenesis, Angiogenesis, and Permeability”, Nantes, F-44000, France

**Keywords:** glioblastoma-initiating cells, chemical screen, apoptosis, self-renewal, temozolomide

## Abstract

Glioblastoma multiforme (GBM) is a highly aggressive tumour of the central nervous system and is associated with an extremely poor prognosis. Within GBM exists a subpopulation of cells, glioblastoma-initiating cells (GIC), which possess the characteristics of progenitor cells, have the ability to initiate tumour growth and resist to current treatment strategies. We aimed at identifying novel specific inhibitors of GIC expansion through use of a large-scale chemical screen of approved small molecules. Here, we report the identification of the natural compound β-escin as a selective inhibitor of GIC viability. Indeed, β-escin was significantly cytotoxic in nine patient-derived GIC, whilst exhibiting no substantial effect on the other human cancer or control cell lines tested. In addition, β-escin was more effective at reducing GIC growth than current clinically used cytotoxic agents. We further show that β-escin triggers caspase-dependent cell death combined with a loss of stemness properties. However, blocking apoptosis could not rescue the β-escin-induced reduction in sphere formation or stemness marker activity, indicating that β-escin directly modifies the stem identity of GIC, independent of the induction of cell death. Thus, this study has repositioned β-escin as a promising potential candidate to selectively target the aggressive population of initiating cells within GBM.

## INTRODUCTION

Glioblastoma multiforme (GBM) is the most frequent primary tumour of the central nervous system (CNS) in adults [1]. GBM is characterised by a diffuse and aggressive phenotype that is associated with rapid cellular proliferation, angiogenesis and necrosis. Current standard treatment for GBM is palliative in nature, typically involving de-bulking surgery followed by radiotherapy and DNA-alkylating chemotherapeutic agents to eliminate the remaining cells. The recent introduction of the Stupp protocol combining radiation therapy with adjuvant chemotherapeutic agent temozolomide (TMZ) has improved GBM 5 year survival from 2% to approximately 10% [2, 3]. However, prognosis for GBM patients remains extremely poor despite these advances, with median survival reported at 14.6 months [2, 4]. The poor long-term survival rates in GBM have been in part attributed to the uncontrolled recurrence of the primary disease following initial therapy.

GBM is associated with significant intertumoural heterogenity and as such the genomic profile of GBM has been well characterised [5]. It is widely recognised that there are 4 subtypes of GBM as defined by their transcriptional profiles; classical, mesenchymal, neural and proneural [6]. The classical GBM subtype is associated with a higher frequency of EGFR mutations and the absence of mutations in TP53 [6]. In contrast, mesenchymal GBM exhibit frequent mutations in NF1, PTEN and TP53 tumour suppressor genes, and correlates with a higher percentage of necrosis and inflammation [6]. Neural GBM shares many of the mutations associated with the other subtypes, but is characterised by a genetic profile more similar to that of normal neurons [6]. The fourth subtype, proneural, features mutations in TP53 as well as frequent mutations in IDH1 and PDGFRA. This subtype occurs particularly in younger patients and is associated with a trend towards prolonged survival compared to the other subtypes of GBM [6]. Interestingly, approximately 75% of lower grade gliomas are identified as proneural and as such are associated with a better prognosis [6, 9]. In contrast, patients with poorer prognosis are found to belong to mesenchymal and classical subsets [9].

Within GBM, a subpopulation of cells with tumour-initiating properties remains largely unaffected by conventional therapies, and as such has been implicated in tumour recurrence [10, 11]. Glioblastoma-initiating cells (GIC) share a number of characteristics with normal neural stem cells such as self-renewal, the ability to migrate and infiltrate the brain parenchyma, as well as the potential for differentiation [12–14]. Additionally, it has been well documented that GIC are more resistant to radiation [15, 16] and chemotherapy [11, 17, 18]. Indeed, GIC possess characteristics that favour the evasion and resistance to current treatment strategies. Chemotherapy and radiotherapy target cycling, highly proliferative cancer cells whereas, GIC are comparatively quiescent and slow cycling and allowed to survive to repopulate the tumour post-treatment [17]. Additionally, the ability of GIC to asymmetrically divide further contributes to the recapitulation of the tumour after treatment. Asymmetric cell divisions allow cells to concurrently self-renew and produce cells destined for differentiation pathways [19, 20]. In the case of GBM, asymmetric cell division following treatment would allow the maintenance of the GIC population, while simultaneously producing more differentiated and proliferative daughter cells to rebuild the tumour. Hence, there is evidence to suggest that current clinical treatments enrich the GIC subpopulation, with the ability to regenerate the tumour. For all these reasons, identifying novel therapeutics that directly targets GIC is of considerable importance in order to improve prognosis for GBM patients.

Considering the potential of anti-GIC therapies to advance GBM treatment, we employed a chemical screen of currently FDA- and EMA- approved small molecules in order to identify a selective inhibitor of GIC expansion with known bio-safety in humans. We conducted this screen in a well-characterised panel of patient-derived, long-term GIC cultures, including GIC derived from each of the four genetic subtypes of GBM [21–26]. These GIC retained the ability to expand *ex vivo* as spheres (tumourspheres, TS) in defined medium, to express progenitor markers, while able to differentiate and also initiate tumour formation experimentally *in vivo* [23, 25, 27]. The cytotoxic effect of the compounds was initially evaluated in two patient-derived GIC growing as spheres and identified β-escin, a mixture of triterpenoid saponins isolated from the seeds of horse chestnuts. Subsequent studies demonstrated the selective inhibition of GIC viability by β-escin, with no toxicity evident in the multiple differentiated GBM, cancer and control cell lines tested. Accordingly, we demonstrated a specific effect of β-escin on induction of apoptosis and modulation of stemness properties of nine individual GIC, indicating the potential of β-escin as a selective and potent inhibitor of GIC.

## RESULTS

### A smart chemical library screen identifies three compounds that are toxic for glioblastoma-initiating cells

**Figure 1:**
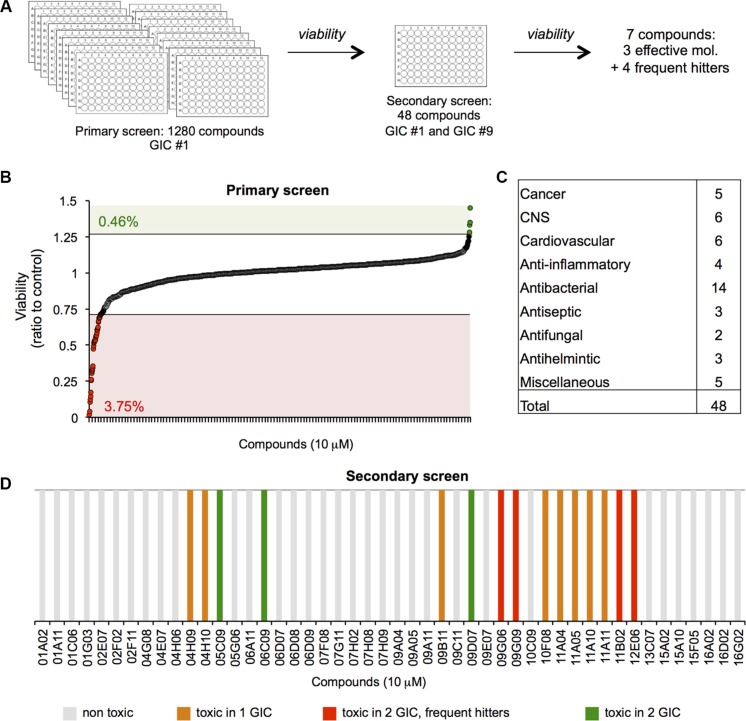
A viability screen identifies three compounds toxic for glioblastoma-initiating cells (**A**) The Prestwick smart chemical library was employed in order to identify molecules with cytotoxic activity in human GICs. (**B**) In the primary screen, 1280 small molecules were tested on mesenchymal GIC#1 and viability assessed at 48 hours. Thresholds were set to detect compounds that induced a potent and significant increase (1.25) or decrease (0.75) in cell viability. Numbers in green and red indicated the percentage of compounds that fell above (0.46%) and below (3.75%) the thresholds. (**C**) Following the primary screen, 48 candidates were identified and tested in both mesenchymal GIC#1 and classical GIC#9. (**D**) In the secondary screen, three molecules were identified that effected viability in both GIC#1 and #9, and were classified as follows: non-toxic (grey bars), toxic in one or the other cell line (orange bars), toxic in the two tested GIC, but frequent hitters (red bars) and toxic in the two tested GIC (green bars).

To identify new compounds with cytotoxic activity in human GIC, we screened a library of 1280 FDA-approved small molecules on one human GIC long-term culture. Cells grown as 3D spheres (tumourspheres, TS) in enriched defined medium were treated with 10 micromolar of each compound, and the fraction of viable cells measured at 48 hours in a 96-well format (Table 1, Figure 1A–1B). Thresholds were set to detect compounds that induced a potent and significant increase (1.25) or decrease (0.75) in cell viability. This led to the identification of 3.75% of candidates from the initial screen as potentially lethal in GIC. A secondary screen conducted in both GIC#1 and GIC#9, from mesenchymal and classical subtypes, respectively (Table 1, Figure 1C) allowed us to exclude 33 molecules, as they exhibited no effect on GIC viability, and were therefore considered as false positives from the primary screen. Four compounds were identified as frequent hitters that previously demonstrated toxicity in other cancer cell lines screened in our laboratory (our unpublished data and [28]). Additionally, eight compounds were cytotoxic in only one of the GIC tested, leaving three remaining molecules that significantly reduced the amount of viable cells in both subtypes of GIC (Figure 1D).

**Table 1:** GIC characterisation

#	Age	Gender	Histology	Sub-Type (Verhaak)	Tumour Initiation	Differentiation
#1	68	M	GBM IV	Mes	+	+
#4	76	F	GBM	Mes	+	+
#5	66	M	GBM IV	Proneural	+	+
#7	49	M	GBM IV	Mes	+	+
#8	79	M	GBM IV	Classic	+	+
#9	68	F	GBM IV	Classic	+	+
#13	59	M	GBM	Neural	+	+
#15	70	F	GBM	Mes	+	+
#16	72	M	GBM IV	Classic	+	+

Tumour initiation was monitored in ectopic xenografts, as described in methods. Differentiation was induced by switching cells from mitogen-containing media to DMEM supplemented with 10% FBS. Differentiation was considered as positive according to morphological changes of adherent cells.

### β-escin is more toxic to glioblastoma initiating cells than perhexiline maleate or chlorprothixene

**Figure 2:**
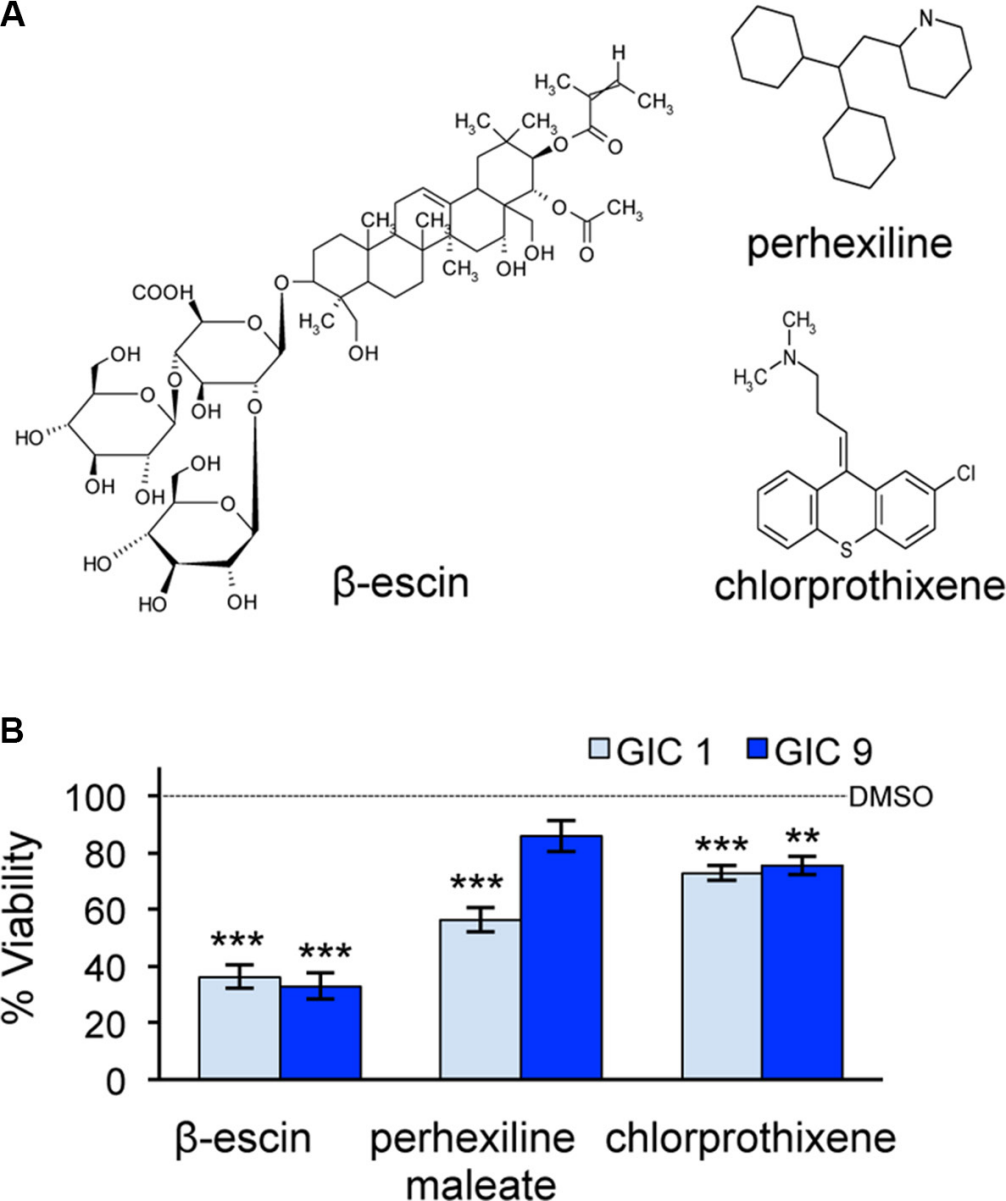
β-escin is more toxic to glioblastoma-initiating cells than perhexiline maleate or chlorprothixene (**A**) Three compounds β-escin, perhexiline maleate and chlorprothixene were identified as potential candidates from the chemical screen. (**B**) Cell viability following treatment with β-escin, perhexiline maleate or chlorprothixene was tested in mesenchymal GIC#1 and classical GIC#9 at 48 hours. Data are representative of 3 independent experiments, each in triplicate **p < 0.01, ***p < 0.001 compared to the DMSO control of each GIC.

The three common hits from the secondary screen were identified as perhexiline maleate, chlorprothixene and β-escin (Figure 2A). A final viability screen was performed on these three compounds in both GIC#1 and GIC#9, using a 24-well format, in order to exclude for a density effect. All three compounds significantly reduced cell viability in GIC#1, however the natural compound β-escin demonstrated the most profound effect in both the GIC tested (Figure 2B). Thus, β-escin emerged as an efficient toxic agent against GIC *in vitro*.

### β-escin selectively targets glioblastoma-initiating cells

**Figure 3:**
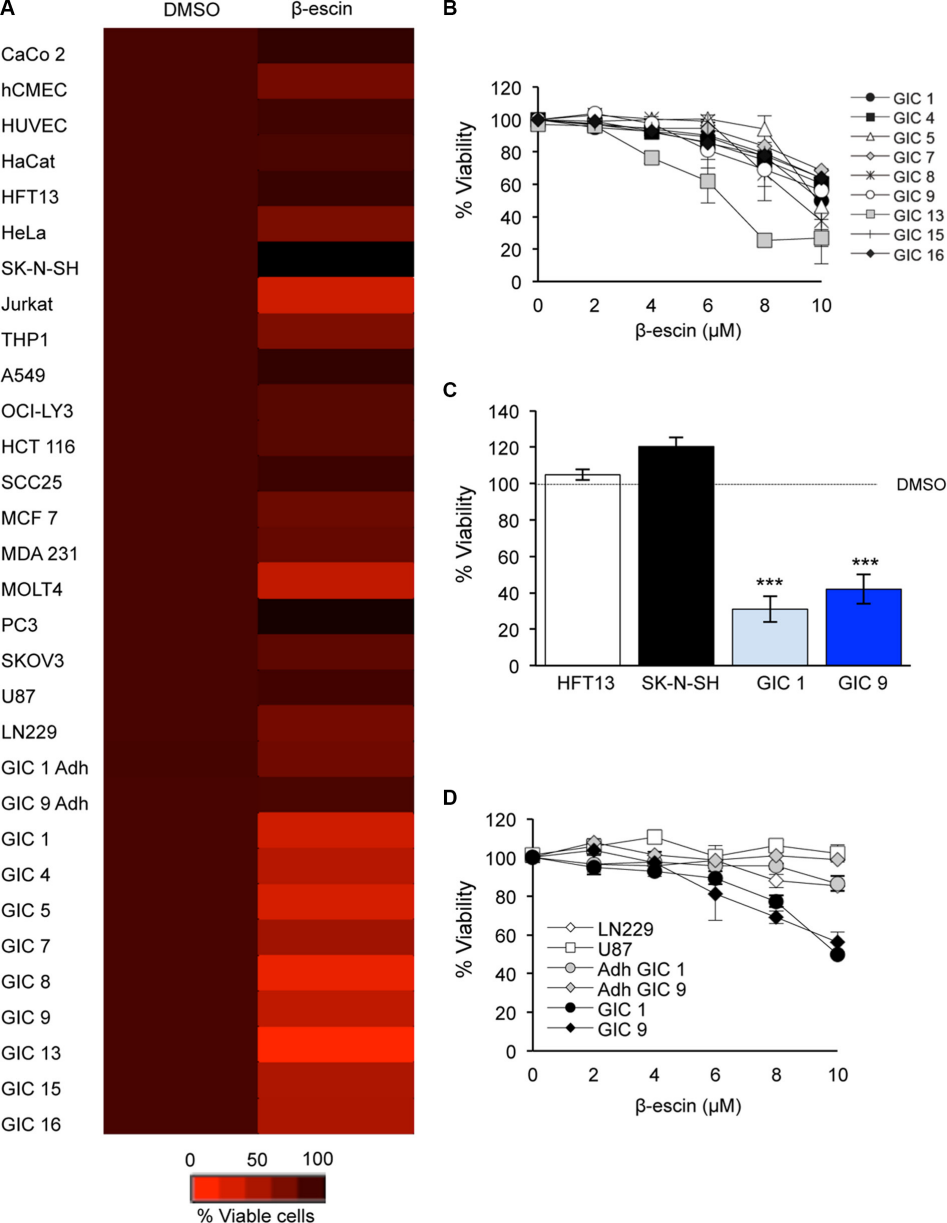
β-escin is a selective inhibitor of glioblastoma-initiating cell viability (**A**) β-escin is not toxic to barely toxic to a panel of 20 human cancer and normal cell lines, and in the adherent differentiated sister cells of mesenchymal GIC#1 and classical GIC#9 (Adh GIC). (**B**) At 48 hours, β-escin reduces GIC viability in a dose dependent manner that is significant at 10 μM (*p < 0.05 for GIC#15, **p < 0.01 GIC#7 and #16, ***p < 0.001 GIC#1, #4, #5, #8, #9 and #13). Please see Table 1 for information on Verhaak molecular subtypes for each GIC. (**C**) Treatment with 10 μM of β-escin does not demonstrate any toxic effect on HTF13 or SK-N-SH. (**D**) β-escin is a selective inhibitor of GIC, and does not affect the viability of GBM lines or the adherent differentiated sister cells of GIC (Adh GIC). Data are representative of 3 independent experiments, each in triplicate, ***p < 0.001 compared to DMSO control for each cell line.

Due to the large scale of the preliminary screening process, no control cell lines were employed. Thus to further investigate the selectivity of β-escin to GIC, we next applied 10 μM of the compound on a panel of 20 human cancer and control cell lines (Figure 3A). This revealed that β-escin did not demonstrate a significant toxic effect in the endothelial, epithelial, keratinocyte or neural cell lines assessed. Furthermore, this dose of β-escin was unable to induce toxicity in the lymphoma, head and neck, primary effusive lymphoma, colorectal, prostate, ovarian, lung or breast cancer cell lines tested. In direct contrast, β-escin significantly reduced viability at this concentration in all 9 GIC tested (Figure 3A). Of note, β-escin diminished the viability of Jurkat and MOLT4 cell lines indicating a potential effect on the T cell lineage, in agreement with previous studies [29]. Hence, our data suggest that β-escin is a selective inhibitor of GIC growth.

In order to determine the optimal dose of β-escin, a dose response assay was performed in nine patient-derived GIC to include the four subtypes of GIC [6] (Table 1, Figure 3B). GIC expansion was reduced following β-escin treatment in a dose-dependent manner, with a significant reduction at 10 μM occurring in all GIC assessed. To more specifically test the effect of β-escin on neural cells, the human adult neural stem cell line HFT13 and human neuronal line SK-N-SH were exposed to 10 μM of the compound in parallel to GIC#1 and GIC#9 for 48 hours (Figure 3C). β-escin demonstrated no significant effect on the percentage of viable neural cells compared to DMSO controls, despite significant toxic effects on GIC at this dose. In order to confirm that β-escin selectively targets GIC, a dose response was performed in the differentiated human primary GBM cell lines, U87, LN229, as well as in GIC#1 and GIC#9 sister cells in which differentiation was induced by addition of serum to the culture media (Figure 3A, 3D) [22, 23, 25]. β-escin no longer induced toxicity in the GBM lines assessed or differentiated GIC at any concentration, further supporting the potential of this compound as a selective inhibitor of GIC maintenance.

### Comparison of β-escin to current standard-of-care

**Figure 4:**
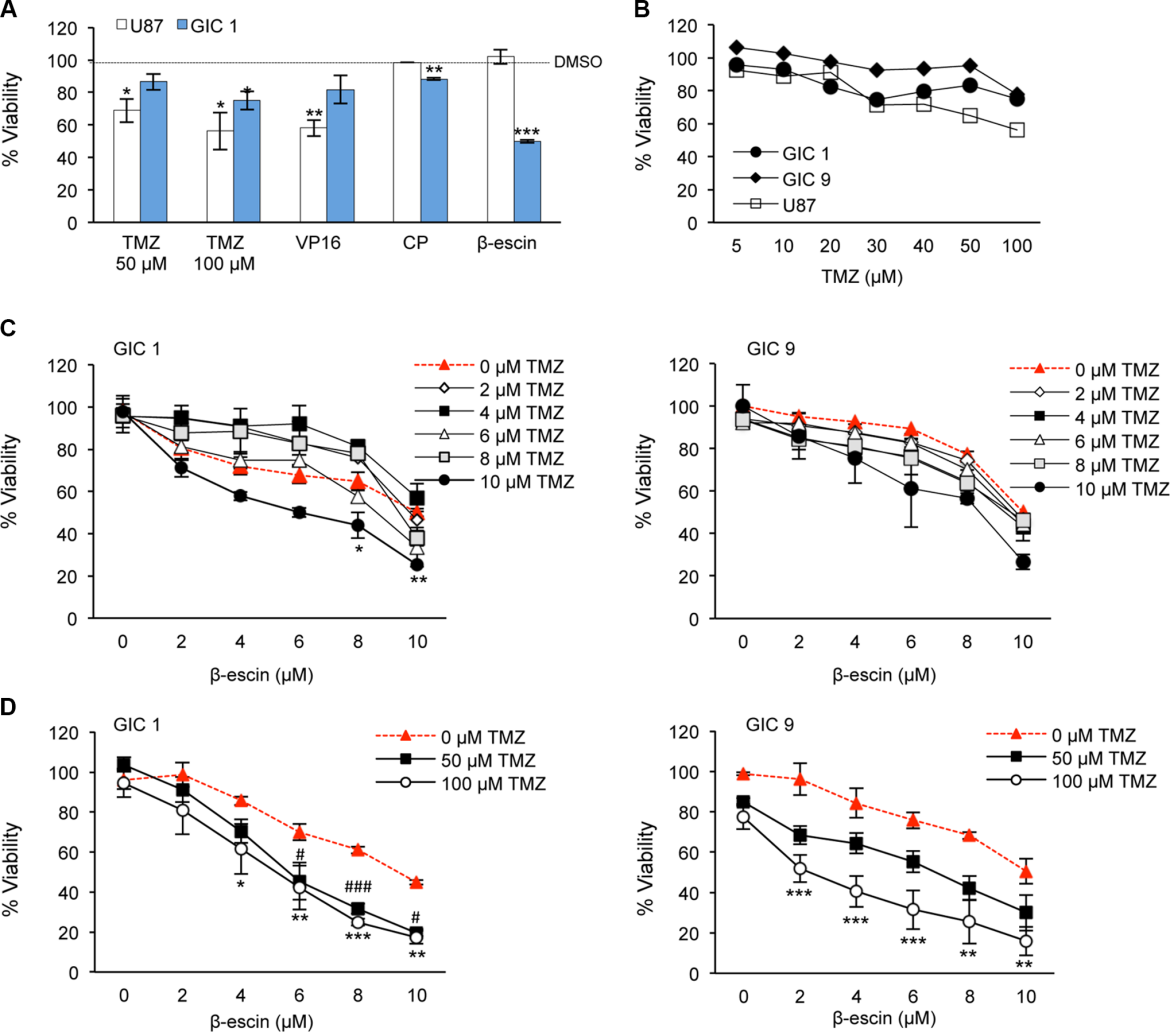
β-escin is a selective inhibitor of glioblastoma-initiating cell viability (**A**) Mesenchymal GIC#1 and GBM line U87 were treated with temozolomide (50 or 100 μM, TMZ), etoposide (20 μM, VP-16), cisplatin (10 μM, CP) or β-escin (10 μM) and toxicity assessed. (**B**) Viability following TMZ treatment was assessed in U87, mesenchymal GIC#1 and classical GIC#9. (**C**) A dose response of β-escin was combined with TMZ to assess the potential combinatory action between these agents in GIC#1 and #9. (**D**) Dose response with higher doses of TMZ compared to β-escin in GIC#1 and #9. Data are indicative of 3 independent experiments, each in triplicate *p < 0.05, **p < 0.01, ***p < 0.001, compared to the DMSO control (A–B) or corresponding dose of β-escin (C–D) for each cell type assessed. ^#^*p* < 0.05, ^###^*p* < 0.001, 50 μM TMZ compared to relative dose of β-escin.

To assess the efficacy of β-escin in comparison to current standard treatments for GBM, GIC#1 and GBM line U87 were treated with temozolomide (TMZ), etoposide (VP-16), cisplatin (CP) or β-escin and viability assessed. Consistent with the literature and chemo-resistant potential of GIC [30], TMZ was more toxic in U87 compared to GIC#1 at both 50 and 100 μM (Figure 4A). Additionally, β-escin was the most effective at significantly reducing cell viability in GIC#1 compared to all other treatments assessed, but demonstrated no effect on U87 viability. Similarly, at 48 hours, the current standard chemotherapeutic agent for GBM, TMZ, demonstrated no overt toxic effect in GIC#1 and GIC#9, but a 20% reduction in cell viability in U87 (Figure 4B). In order to examine the potential of combined therapy with β-escin and TMZ, a dose response using low doses of TMZ in combination with β-escin was performed. In both GIC examined, the addition of TMZ resulted in a dose-dependent effect on cell viability compared to β-escin alone that was significant for GIC#1 at the highest combined doses (Figure 4C). Furthermore, at higher doses of TMZ (50 and 100 μM) both GIC exhibited further reductions in cell viability that was significant even at low doses of β-escin (Figure 4D). These results suggest that β-escin may chemosensitize GIC to TMZ, and raises the possibility of a beneficial adjuvant therapeutic action.

### β-escin induces apoptosis in glioblastoma-initiating cells

**Figure 5:**
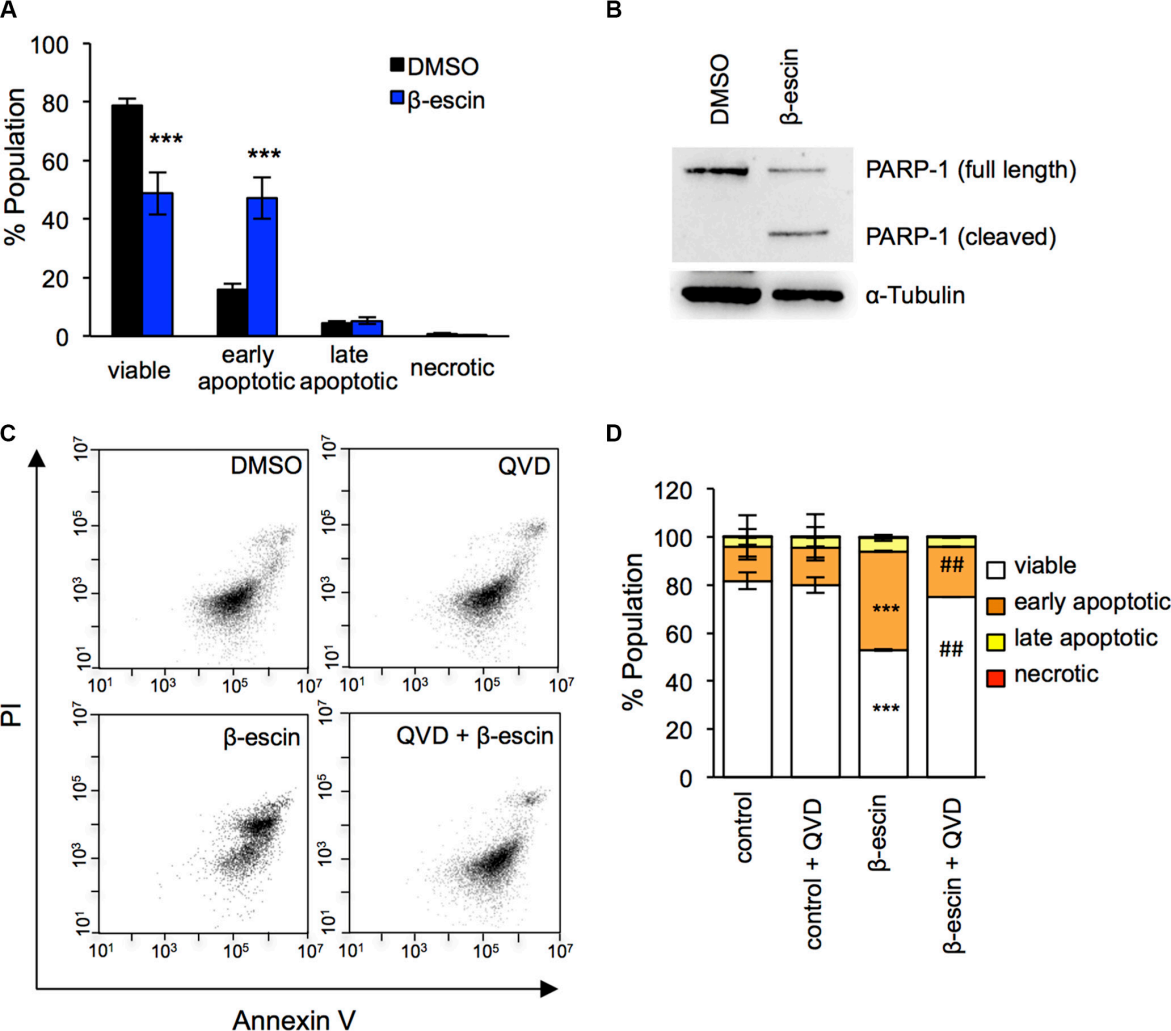
β-escin induces apoptosis in glioblastoma-initiating cells (**A**) Administration of 10 μM β-escin significantly reduced the number of viable mesenchymal GIC#1, and increased early stage apoptosis compared to DMSO treated controls. (**B**) PARP-1 cleavage was evident 24 hours following β-escin treatment. (**C**–**D**) Pre-treatment with the pan-caspase inhibitor QVD prior to β-escin administration rescued β-escin induced apoptosis. Data are representative of 3 independent experiments, each in triplicate ***p < 0.001 compared to DMSO control, ^##^*p* < 0.01 compared to β-escin treatment.

In order to further evaluate how β-escin selectively reduces GIC expansion, cell death was assessed with Annexin V/Propidium Iodide (PI) staining by flow cytometry in GIC#1. In keeping with the Uptiblue viability assay results, administration of β-escin significantly increased the number of apoptotic cells compared to DMSO treated controls, while the percentage of necrotic cells remained even (Figure 5A). Consistent with these findings, we observed a robust cleavage of the caspase substrate PARP-1, 24 hours following β-escin treatment (Figure 5B). Finally, pre-treatment of cells with the pan-caspase inhibitor QVD prior to β-escin administration nearly entirely abolished cell death (Figure 5C–5D). Overall, this data demonstrates that β-escin kills GIC through caspase-dependent cell death.

### β-escin alters the stemness properties of glioblastoma-initiating cells

**Figure 6:**
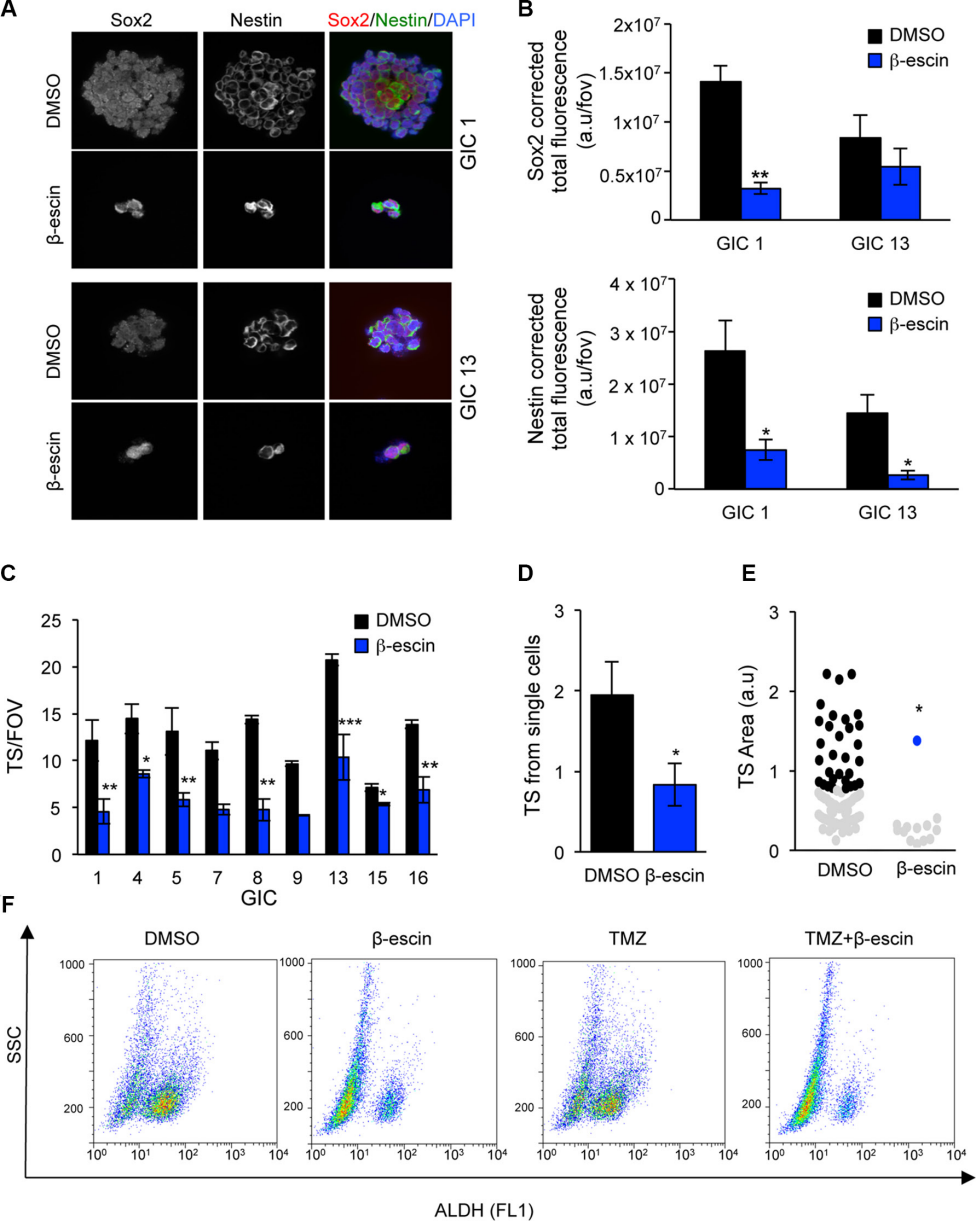
β-escin alters the stemness properties of glioblastoma-initiating cells (**A**–**B**) SOX-2 and Nestin expression were assessed following β-escin treatment using confocal microscopy and total corrected fluorescence calculated in mesenchymal GIC#1 and neural GIC#13. (**C**) Administration of 10 μM of β-escin impaired TS formation in nine human GIC lines assessed as represented as the number of tumoursphere per five fields of view (TS/FOV). (**D**) GIC#1 was seeded at a density of 1 cell per well the self-renewal ability assessed at day 7. (**E**) β-escin induced decreases in TS size in GIC#1. (**F**) Analysis of β-escin and TMZ induced changes to ALDH activity by flow cytometry. Data are representative of 3 independent experiments, *p < 0.05, **p < 0.01, ***p < 0.001 compared to DMSO controls.

We next investigate the effect of β-escin on the stem properties of GIC. Firstly, our confocal analysis of the immunofluorescence staining for the stemness markers SOX-2 and Nestin revealed a significant decrease in these markers in both mesenchymal GIC#1 and neural GIC#13, following β-escin treatment (Figure 6A–6B). A similar impairment in tumoursphere (TS) formation was observed in seven additional human GIC lines, covering the four molecular GBM subtypes (Figure 6C, Table 1) as well as a significant reduction in the self-renewal capabilities and TS size of GIC#1 (Figure 6D–6E). Consistent with these results, β-escin notably reduces the percentage of stem marker, ALDH, compared to DMSO controls. Conversely, no changes to the ALDH population were observed following TMZ treatment, with the β-escin induced decrease in ALDH activity maintained when combined with TMZ (Figure 6F). These results suggest that β-escin significantly alters the stem profile of GIC.

### Preventing apoptosis does not halt β-escin action on glioblastoma-initiating cells

**Figure 7:**
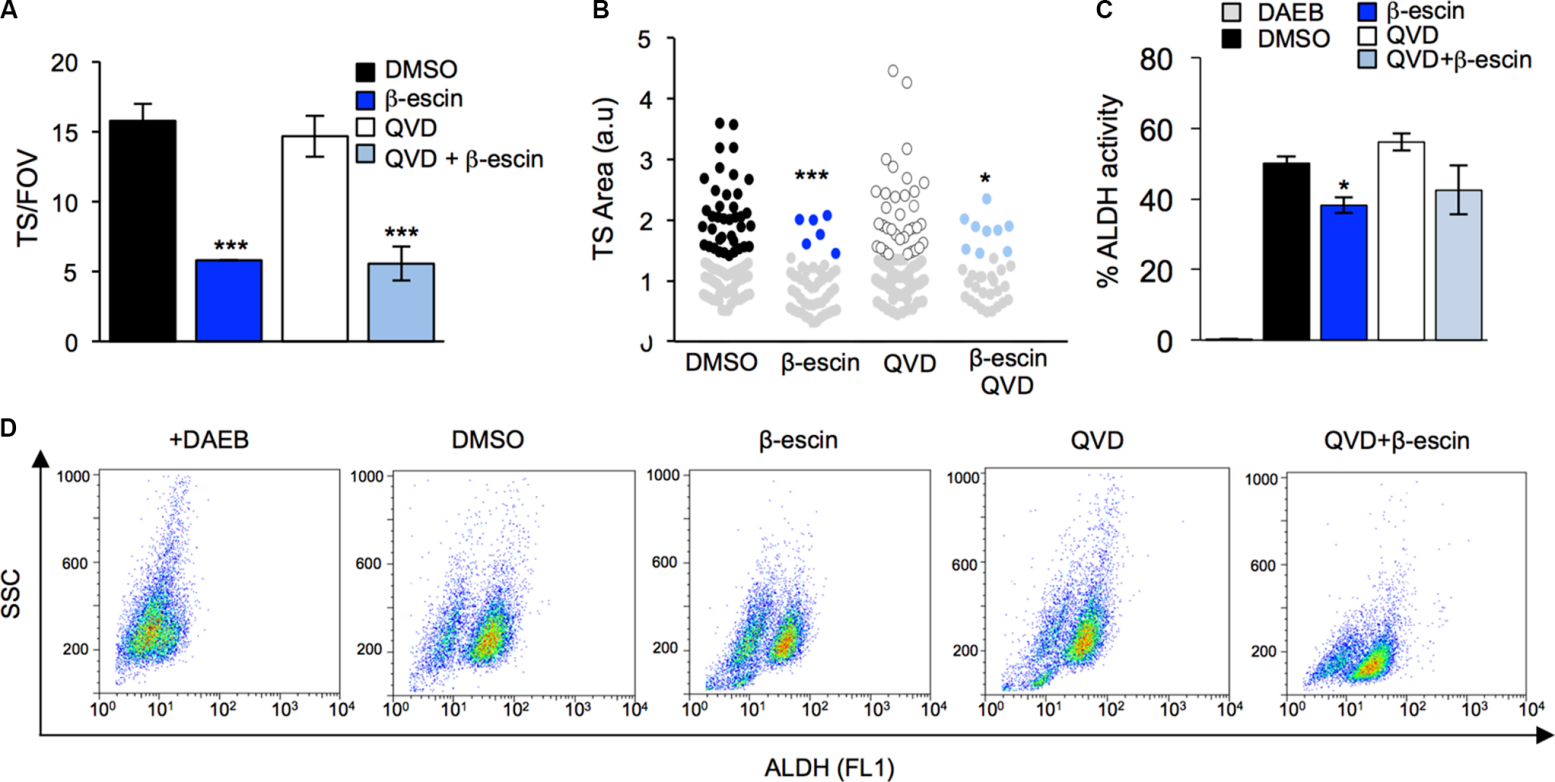
Preventing apoptosis does not halt β-escin action on glioblastoma-initiating cells (**A**) Pre-treatment of mesenchymal GIC#1 with the caspase inhibitor QVD does not alter β-escin induced decreases in TS formation. (**B**) TS size in response to QVD and β-escin treatment. (**C**–**D**) Addition of QVD in combination with β-escin did not rescue the β-escin induced reduction in TS formation or ALDH activity. Data are representative of 3 independent experiments, *p < 0.05, **p < 0.01, ***p < 0.001 compared to DMSO controls.

To next assess whether the effect of β-escin on TS maintenance is a direct consequence of cell death, QVD was administered in combination with β-escin and TS formation assessed. Importantly, blocking caspase-dependent apoptosis with QVD did not prevent β-escin from reducing TS formation in GIC#1 (Figure 7A). Similarly, TS size was significantly reduced following β-escin treatment, and preserved following pre-treatment with QVD (Figure 7B). Consistent with this, the percentage of cells exhibiting high levels of activity of ALDH was reduced in GIC#1 following β-escin treatment compared to DMSO and remained lower when β-escin was combined with QVD (Figure 7C–7D). Taken together these results indicate that the effect of β-escin on GIC maintenance is not a passive consequence of cell death. Instead, our data strongly support the hypothesis that β-escin selectively targets the stemness identity of GIC, independent of the induction of cell death.

## DISCUSSION

Despite considerable research focus and accelerated procedures for clinical trials, survival rates for GBM remain extremely poor. Glioblastoma-initiating cells (GIC) have been implicated in GBM initiation, as well as resistance to current therapies and tumour recurrence following treatment. As such, they represent a promising cellular target to improve outcome for GBM patients. This study sought to identify and reposition a new compound that could selectively target the initiating population of cells within GBM. We employed a chemical screen of chemically and pharmacologically diverse small molecules with known efficacy and safety in humans. β-escin demonstrated significant toxicity in GIC, but not in a panel of human cancer and normal cell lines, suggesting it selectively targets GIC. Moreover, it does not reduce the expansion of more differentiated GBM cells, such as cell lines or differentiated adherent sister GIC. This natural compound was more effective than current cytotoxic anti-cancer drugs; including temozolomide (TMZ), etoposide (VP16) and cisplatin, in reducing GIC viability. Additionally, combined treatment of TMZ and β-escin resulted in a significant reduction in cell viability that was greater than either treatment alone. From a molecular standpoint, we found that β-escin reduces cell viability via the induction of apoptosis, which can be halted by the pan-caspase inhibitor QVD. However, QVD was unable to rescue β-escin-induced changes in the stem properties of GIC, suggesting that β-escin alters the stemness of GIC independent of the induction of apoptosis. Our results thus indicate that β-escin is a potential candidate to specifically target GIC within GBM.

Our study identifies β-escin as a potent and selective inhibitor of GIC viability. β-escin is a natural compound isolated from the seeds of the *Aesculus hippocastanum* (horse chestnut) plant. Escin is a natural mixture of triterpene saponins that has two forms, α and β, of which β-escin is the active component [31]. It has been well established that this plant extract has potent anti-inflammatory, anti-edematous as well as analgesic properties and consequently has yielded positive results as a treatment for post-operative edema and chronic venous insufficiency [31–37]. In recent years, multiple studies have identified β-escin as a potential anti-cancer agent. Similar to our results in Jurkat and MOLT4 cell lines, Zhang and colleagues reported that β-escin induces apoptosis in acute leukaemia T cells via induction of the intrinsic cell death pathway [29]. In addition, β-escin has been reported to induce apoptosis in a number of cancer cell lines, including pancreatic carcinoma [38], lung adenocarcinoma [39], cholangiocarcinoma [40, 41] and gastric adenocarcinoma [42] via a reduction in cellular proliferation and induction of apoptosis. However, we did not detect any obvious action of β-escin on more differentiated human tumour cell lines *in vitro*; a result, which differs from previous studies and may be due to the possible heterogeneity and percentage of tumour initiating cells in cell culture.

Despite the effect of β-escin on proliferation and subsequent induction of cell death indicated in multiple cell lines, there is currently no consensus on the mechanism of action. It has been reported that β-escin induces cancer cell death through the mitochondrial caspase dependent pathway [41], inhibition of NF-κB signalling [36, 38] and GSK3β/β-catenin pathway [40] indicating that the mechanism of action may be cell type dependent. As such, further analysis is required to clearly decipher the mode of action of β-escin in GIC. Preliminary experiments from our laboratory have nonetheless excluded both a possible transcriptional effect of β-escin, as a gene array analysis did not return any validated hits, and the possibility that the compound exerts a paracrine action with β-escin conditioned culture medium not sufficient to alter GIC viability (our unpublished results). Nonetheless, our data clearly indicate that β-escin potently reduces GIC viability, induces apoptosis, and specifically modifies the stem properties of GIC.

Our observation that pre-treatment with a pan-caspase inhibitor is able to inhibit apoptosis but not alterations to self-renewal support the hypothesis that β-escin directly targets the stem properties of GIC, independent of the induction of apoptosis. Previous studies reported that β-escin is protective against hypoxia-induced alterations [43, 44]. It is well documented that GIC reside in hypoxic niches, which maintains GIC in a quiescent state, and may protect the GIC from chemo- and radiation therapy [45, 46]. As such, alterations in this hypoxic microenvironment may make the GIC more susceptible to apoptosis and conventional therapies; a possible action of β-escin in this context will need to be further challenged. Interestingly, not only did co-treatment of β-escin with the pan-caspase inhibitor QVD result in alterations to stem activity, the combination of these compounds also appears to impact on SSC in the ALDH assay. Loss of cell volume is a well-documented early feature of apoptosis, with alterations in cell size reported to occur independently of caspase activation in response to certain apoptotic stimuli. Indeed, it has been described that treatment with apoptotic agents in the presence of pan-caspase inhibitors have failed to inhibit apoptotic cell shrinkage [47–49] in some cell lines. Thus, the observed effect on granularity in the present study may reflect initiation of the early stages of apoptotic cascade upon β-escin stimulation that occurs upstream of activation of the caspases.

Along with other stemness markers, aldehyde dehydrogenases (ALDH) have been used to isolate stem like cells [50]. ALDH belongs to a family of intracellular enzymes that play an important role in cellular oxidative processes, including chemoresistance. In GBM, high ALDH1A1 expression is associated with poor prognosis and its overexpression *in vitro* a predictor of TMZ resistance [51]. Moreover, enrichment of *ALDH* family genes in GIC has been observed [52]. In the present study, β-escin treatment decreased the percentage of ALDH activity in GIC; consistent with previous reports that β-escin inhibits ALDH activity in H460 human lung cancer cells [39]. Furthermore, we demonstrate that treatment with TMZ alone has no effect on ALDH activity, and when co-administered with β-escin does not alter the β-escin induced decrease in ALDH positive cells. As ALDH1A1 has been linked to the resistance of cancer stem cells to chemotherapy, compounds that alter ALDH activity may present an opportunity to target tumour populations resistant to current chemotherapeutic agents.

The ineffectiveness of chemotherapy in GBM has in part been attributed to GIC within the tumour. Consequently, agents that sensitize GIC to chemotherapeutic agents are of great interest to improve prognosis for GBM patients. Combination therapies in GBM have numerous advantages, particularly as combining drugs that work by different mechanisms or target specific populations of cells such as GIC, may achieve greater therapeutic results than either drug alone. Furthermore, the dose of cytotoxic drugs such as TMZ cannot be limitlessly increased without associated increases in adverse side effects [53]. Thus, combination therapy presents the opportunity to achieve higher therapeutic efficacy at lower drug doses. Our results indicate that combined treatment with β-escin significantly improves TMZ action in GIC. Indeed, previous studies have indicated that β-escin significantly reverses drug resistance [38, 40]. In the present study, we observed differences in the degree of sensitivity to combined treatment between GIC#1 and GIC#9, with significant reductions in cell viability only observed in GIC#9 at 100 μM of TMZ. Given these two GIC are derived from mesenchymal and classical GBM respectively, these results may indicate that sensitivity to TMZ is subtype dependent and should be explored further in subsequent studies. Nonetheless, the significant reduction in GIC viability with combined treatment supports the potential of β-escin as an adjuvant therapy in GBM.

The results of this study strongly suggest that β-escin is a promising potential therapeutic for the selective inhibition of GIC. In addition, β-escin is a currently approved and safety-tested natural compound for clinical use in a number of disorders. In order to further investigate the potential of β-escin as a therapy for GIC, more in-depth pharmacological analysis is required to determine the efficacy of β-escin across the blood brain barrier (BBB), as well as *in vivo* analysis of the effect of β-escin on GIC both alone and in combination with TMZ.

In summary, these data establish the efficacy of β-escin as a selective inhibitor of GIC maintenance *in vitro*. β-escin was able to induce apoptosis in GIC following a reduction in self-renewal and ALDH activity. Furthermore, β-escin appears to potentiate TMZ action on GIC, resulting in a greater effect on viability than either drug alone. Accordingly, β-escin warrants consideration for further development as a selective and adjuvant treatment for GBM.

## MATERIALS AND METHODS

### Ethical statement

Prior to sample collection for diagnostic purposes, informed written consent was obtained from all patients. This study was approved by the respective institutional ethics committees (Sainte Anne Hospital, Paris, France and Laennec Hospital, Nantes, France) and abides by the rules of the Helsinki Protocol.

### Pharmacological agents

For the initial screen, a smart chemical library was obtained from Prestwick Chemicals (http://www.prestwickchemical.com/prestwick-chemical-library.html). The library consisted of 1280 small molecules and natural compounds approved by the FDA, EMA and other agencies. These compounds were chemically and pharmacologically diverse with known efficacy and bio-safety in humans. To confirm the effect of β-escin observed in the screen, subsequent studies utilised β-escin from an alternate source (Sigma Aldrich, E-1378). The pan caspase OPH inhibitor, QVD was used to inhibit apoptosis (R&D systems). To compare Escin to current chemotherapeutic agents, Temozolomide (TMZ, 50–100 μM, Sigma-Aldrich), Etoposide VP16 (20 μM, Sigma-Aldrich) and Cisplatin (10 μM, Tocris) were used.

### Cell culture

The patient derived GIC utilised in this study have been chosen so all four subtypes are represented (Table 1). In particular, we have focused on mesenchymal GIC#1 and classical GIC#9. These two subtypes have been associated with a poorer prognosis when compared to the other GBM subsets [9]. GIC were isolated from patients as previously described [21, 22, 24–27]. Briefly, tumour samples were gently dissociated using the MACsDissociator (Miltenyi) and the initiating cell population characterised by their self-renewal properties, cell surface antigens, genetic signature and ability to differentiate [21] (Table 1). GIC and HTF13 neural stem cells (a gift from PO Couraud, Institut Cochin, Paris, France) [54] were maintained as spheres in DMEM/F-12 and 1% penicillin and streptomycin (PS) (Gibco) with N2, G5 and B27 supplements (Life Technologies) or Neurobasal media (Life technologies) supplemented with 1% PS, 1% glutamax, B27 supplement and heparin (8 μg/ml), with additional growth factors bFGF (20 ng/ml) and LIF (10 ng/ml) added extemporaneously, respectively. To induce differentiation in GIC, these three supplements were omitted and 10% fetal calf serum (FCS) added to the medium.

All cell lines were purchased from ATCC (LGC Standards) or DSMZ. U87-MG and SK-N-SH was cultured in MEM (Gibco), 10% FCS and 1% PS. LN229, HCT-116, SCC25, HUVEC, HeLa and HaCat cells were maintained in DMEM (4.5 g/L glucose, Gibco), 10% FCS and 1% PS. A459, MCF-7, SKOV3, MOLT4, Jurkat, THP1 and MDA-231 cell lines were cultured in RPMI (Gibco), 10% FCS and 1% PS. Similarly, OCI-LY3 and BC3 were maintained in RPMI with 20% FBS, 1% PS. CaCo2 cells were cultured in DMEM (4.5 g/L glucose, Gibco), 20% FCS and 1% PS. Prostate cancer cell line PC3 was cultured in DMEM F12, with 1% glutamax, 1% PS and 10% FBS. Immortalized human cerebral microvascular endothelial cells (hCMEC [25]) were cultured in Endothelial Basal Medium-2 (EBM-2, Lonza), containing 5% FBS, 1% P/S, HEPES, hydrocortisone (1.4 mM), ascorbic acid (5 mg/ml) and basic fibroblast growth factor (1 ng/ml) (Sigma).

### Cell viability assays

Cell viability in suspension cells was tested using the UptiBlue reagent (Interchim), a flurometric/colourmetric growth indicator based on the detection of metabolic activity. Optimal seeding density was determined in a pilot study, and 2 × 10^3^ cells per well were plated in a 96 well plate in triplicate and the relevant treatment administered. UptiBlue was added at a concentration of 10% v/v and cells maintained at 37°C 5% CO_2_ until analysis. Absorbance was measured 48 hours following treatment at 570 and 600 nm on a FluStar Optima (BMG Labtech) plate reader, and the percentage of cell viability calculated relative to the vehicle control conditions, according to the manufacturers instructions.

Cell survival in adherent cells was evaluated using the MTT assay (1-(4,5-dimethylthiazol-2-yl)-3,5- diphenylformazan, thiazolyl blue formazan, Sigma-Alrich), which is reduced to formazan based on the mitochondrial activity of living cells. Cells were seeded in a 96-well plate in triplicate at a density of 5 × 10^3^ per well and treatments administered 24 hours after seeding. The day of analysis, cells were incubated with MTT (25%, v/v) in culture medium for four hours following which formazan crystals were dissolved in 100 μL of DMSO. Absorbance values were read at 590 nm and expressed as a percentage of cell viability relative to basal conditions.

### Tumoursphere formation assay

To test the effect of β-escin on tumoursphere (TS) formation, 100 GIC/μL were plated in triplicate in sphere maintaining media as previously described [23]. Cells were manually dissociated each day and a single cell suspension maintained for 3 more days. TS were counted in five random fields of view (fov), and the mean from the triplicate of each condition calculated from three independent experiments.

In order to examine the effect of β-escin on pure self-renewal, GIC#1 was seeded at a density of 1 cell per well in a 96 well plate, and the number of TS per well counted at 7 days.

To calculate the TS size in response to treatment, GIC#1 were plated and maintained as described for the TS assay. Representative images for each well were taken on a light microscope for analysis. TS area was calculated from 5 images per well for each treatment, using ImageJ (v1.48, NIH) software.

### Flow cytometry

To assess apoptotic populations following β-escin treatment, the AlexaFluor 488 Annexin V/Dead cell Apoptosis Kit was used (Life Technologies). Briefly, following 48 hours of β-escin, QVD or combined treatment, cells were harvested, washed in cold PBS and incubated with Annexin V/ PI probes for 15 minutes at room temperature. Following the incubation period, binding buffer was added to the samples on ice. Sample analysis was performed on the Accuri (BD Biosciences) and analysed using the associated software (CFlow plus, BD Bioscience).

Analysis of ALDH activity was performed using the ALDEFLUOR assay kit (Stem cell technologies). Briefly, cells were incubated with ALDEFLUOR or in combination with an ALDH activity inhibitor (DAEB) at 37°C for 45 minutes. Samples were analysed on FACSCalibur (BD Bioscience) according to the manufacturers instructions.

### Immunofluorescence

In preparation for immunofluorescence staining, TS were dropped on poly-lysine coated slides (Thermo Fisher Scientific) and fixed in 4% PBS paraformaldehyde. Immunofluorescence was performed as previously described [23], cells were incubated with SOX-2 or Nestin (Millipore) primary antibodies, followed by fluorescent conjugated secondary antibodies (Life Technologies) and mounted with DAPI-containing mounting medium (Life Technologies). Confocal images were acquired on the TCS/SP5 Leica confocal microscope (Institut Cochin, Paris).

To calculate the total corrected fluorescence (TCF) of SOX-2 and Nestin we used ImageJ (v1.48, NIH). An outline was drawn around each TS and area, mean fluorescence and several adjacent background readings measured. The total corrected cellular fluorescence (TCCF) = integrated density – (area of selected cell × mean fluorescence of background readings), was calculated as previously described [55].

### Western blot

Following stimulation with the relevant treatment, cells were collected and washed in PBS before lysis at 4°C with TNT buffer (50 mM Tris pH 7.4, 150 mM NaCl, 2 mM EDTA, 1% Triton X-100, 1% Igepal) supplemented with protease inhibitors (Sigma-Aldrich). Equal amounts of protein were loaded on Tris-glycine gels and transferred onto nitrocellulose membranes (GE Healthcare). Antibodies against PARP-1 and α-Tubulin (Santa Cruz) were incubated with the membrane overnight at 4°C and followed by incubation with the relevant secondary antibodies (Southern Biotech) for one hour at room temperature. Membranes were revealed using a chemi-luminescent HRP substrate (Millipore) and visualised using the Fusion imaging system (Vilber Lourmat).

### Statistical analysis

All data are representative of three independent experiments unless otherwise stated, and expressed as mean ± SEM. Data were analysed using a one-way or two-way analysis of variance (ANOVA), followed by Bonferroni multiple comparisions tests or unpaired *t*-tests, as appropriate. A *p value* of less than 0.05 was deemed significant in all experiments.
